# Effects of folic acid with vitamin B12/vitamin B6 intervention on serum homocysteine metabolism and complications in patients with type 2 diabetes: a systematic review and meta-analysis of randomized controlled trials

**DOI:** 10.3389/fnut.2025.1701310

**Published:** 2025-11-28

**Authors:** Yaqi Yin, Ziming Zhao, Xu Wang, Qibiao Wu

**Affiliations:** 1 Faculty of Chinese Medicine and State Key Laboratory of Mechanism and Quality of Chinese Medicine, Macau University of Science and Technology, Taipa, Macao SAR, China; 2 Zhuhai M.U.S.T. Science and Technology Research Institute, Guangdong-Macao ln-Depth Cooperation Zone in Hengqin, Zhuhai, Guangdong, China; 3 The First Clinical Medical College, Nanjing University of Chinese Medicine, Nanjing, China

**Keywords:** folic acid, B vitamins, type 2 diabetes mellitus, homocysteine, diabetic complications, meta-analysis

## Abstract

**Aims:**

Folic acid and B vitamins play key roles in regulating serum homocysteine metabolism. Elevated homocysteine levels have been associated with insulin resistance and diabetes-related complications in patients with type 2 diabetes mellitus. This study evaluated the effects of folic acid supplementation combined with B vitamins (B12 and B6) on homocysteine levels and complication risk in adults with type 2 diabetes mellitus.

**Methods:**

We conducted a systematic search of eight databases from inception through August 30, 2025. The primary efficacy outcome was serum homocysteine level, while safety outcomes included the overall incidence and specific types of diabetes-related complications. Meta-analyses were performed using R version 4.5.0 with the meta package (version 8.1-0), employing random-effects models. Results are presented as standardized mean differences (SMDs) with 95% confidence intervals (CIs) for continuous outcomes and risk ratios (RRs) with 95% CIs for dichotomous outcomes.

**Results:**

Twenty-nine studies met the inclusion criteria: 26 investigated folic acid plus vitamin B12, one examined folic acid plus vitamin B6, and two evaluated folic acid in combination with both vitamins B12 and B6. Supplementation with folic acid plus vitamin B12 (SMD = −2.77, 95% CI [−3.23, −2.30], *P* < 0.0001) significantly reduced serum homocysteine levels and decreased the incidence of total complications (RR = 0.30, 95% CI [0.24, 0.38], *P* < 0.0001). Similar results were also observed for specific complication subtypes.

**Conclusions:**

Supplementation with folic acid and vitamin B12 may reduce homocysteine levels and the risk of complications in patients with type 2 diabetes mellitus. The substantial heterogeneity and limited sample size of our results necessitate confirmation through additional high-quality, large-scale randomized controlled trials.

**Systematic review registration:**

https://www.crd.york.ac.uk/PROSPERO/view/CRD420251141157.

## 1 Introduction

Diabetes mellitus (DM) is a complex chronic metabolic disease characterized by abnormal glucose metabolism, endocrine dysfunction, and associated complications. Globally, DM affected 537 million adults in 2021, and this number is projected to rise to 783 million by 2045 (1). Type 2 diabetes mellitus (T2DM), which accounts for nearly 95% of cases (2), primarily results from insulin resistance (IR) and impaired insulin secretion from pancreatic β-cell dysfunction. The occurrence of acute complications is closely linked to high mortality rates, whereas chronic complications arise from prolonged hyperglycemia. Microvascular complications include diabetic retinopathy, nephropathy, and neuropathy, whereas macrovascular complications involve cardiovascular and cerebrovascular diseases. These complications reduce patients' quality of life and life expectancy, imposing a significant burden on patients, families, and healthcare systems.

Elevated homocysteine (HCY) levels in T2DM patients are an independent risk factor for early mortality (3) and may play a causal role in T2DM development, as supported by Mendelian randomization trials (4). Disruption of HCY metabolic pathways leads to its excessive accumulation, contributing to vascular and cellular damage. T2DM patients experience exacerbated HCY metabolic abnormalities due to IR and metabolic disturbances. Elevated HCY levels accelerate DM progression and worsen complications such as cardiovascular diseases (CVD) and kidney damage (5). Folic acid deficiency is strongly associated with elevated HCY levels, increasing CVD risk in T2DM patients (6). Long-term metformin use may interfere with vitamin B12 absorption and disrupt folic acid metabolism, reducing the levels of these vitamins (7). This reduction contributes to elevated HCY levels and increases the risk of CVDs and neuropathy (8).

Although many randomized controlled trials (RCTs) have examined folic acid supplementation alone or with B vitamins in individuals with T2DM, most have focused on glycemic indices or diabetes risk, with HCY levels often being a secondary outcome or not assessed. The combination of folic acid and B vitamins, especially vitamins B6 and B12, effectively reduces HCY concentrations (9). However, a randomized controlled crossover trial reported no significant difference in HCY reduction between the folic acid group and the placebo group (10). Mangoni et al. (11) reported that folic acid supplementation significantly reduced serum HCY levels but did not improve lipid or glycemic parameters in T2DM patients.

Existing meta-analyses have focused on the effects of folic acid supplementation on glycemic control (12–15) but have largely excluded studies primarily investigating HCY. This gap highlights the need for an updated and focused meta-analysis. To address this gap, we conducted a systematic review and meta-analysis of RCTs to evaluate the effect of folic acid and B vitamins supplementation on HCY levels in adults with T2DM. This study aims to resolve prior inconsistencies in the literature and provide a stronger evidence base for clinical practice. Additionally, this study explored the potential role of folic acid plus B vitamins in reducing T2DM-related complications.

## 2 Materials and methods

### 2.1 Databases and search strategy

This study adhered to the 2020 Preferred Reporting Items for Systematic Reviews and Meta-Analyses (PRISMA 2020) (Supplementary Table S1) guidelines (16) and was registered in PROSPERO (CRD420251141157) on 19th of February 2025. Any deviation from the protocol has been recorded in Supplementary Table S2. Two independent reviewers (Yaqi Yin and Xu Wang) systematically searched the following databases: PubMed, Web of Science, Embase, the Cochrane Library, China National Knowledge Infrastructure (CNKI), Wanfang Data, China Science and Technology Journal Database (VIP), and the Chinese Biomedical Literature database (CBM). To ensure comprehensiveness, supplementary manual searches were performed, and reference lists of relevant articles were reviewed to identify additional RCTs. The search covered the period from database inception to August 30, 2025.

The English database was searched via the following search formula: (type 2 diabetes mellitus OR T2DM OR type 2 diabetes OR type II diabetes mellitus OR diabetes mellitus type 2) AND (folic acid OR folate OR folacin OR vitamin B9 OR vitamin M OR 5-methyltetrahydrofolate OR pteroylglutamic acid) AND (vitamin B6 OR pyridoxal 5′-phosphate OR pyridoxine OR vitamin B12 OR cobalamin OR methylcobalamin). The complete search strategies for each database are provided in Supplementary Table S3. Language and publication type were not restricted during the search. All the studies that met the inclusion criteria were added to Endnote software for screening.

### 2.2 Inclusion and exclusion criteria

The RCTs included in this study were required to meet the following criteria: (1) Participants had T2DM; (2) Participants were adults aged >18 years; (3) Participants had a T2DM diagnosis confirmed by clinical symptoms and laboratory findings, including fasting blood glucose ≥7.0 mmol/L, postprandial blood glucose ≥11.1 mmol/L, or glycated hemoglobin ≥6.5%; (4) All patients with type 2 diabetes received the same background therapy, such as metformin or other glucose-lowering medications; (5) The intervention group received folic acid combined with vitamin B12 and/or vitamin B6 in addition to the background therapy; (6) The control group either received background therapy alone or background therapy plus placebo.

Studies were excluded if they met one of the following criteria: (1) Reviews, meta-analyses, overviews; (2) observational studies or non-human studies; (3) Lack of data on outcome indicators. Studies meeting any of these criteria were excluded from the analysis.

### 2.3 Data extraction

On the basis of the preset inclusion and exclusion criteria, two independent reviewers screened all the retrieved literature and finalized the eligible studies. Data were independently extracted by the reviewers and included the first author's name, year of publication, country, disease type, mean age, total sample, interventions, controls, presence of comorbidities, and types of comorbidities (Table 1). To ensure the comprehensiveness of the RCTs screening and data accuracy, any disagreements between the two reviewers (Yaqi Yin and Xu Wang) were resolved through discussion with a third independent reviewer (Qibiao Wu), who helped reach a consensus.

**Table 1:** Baseline characteristics of the included studies.

**Studies**	**Country**	**Age (T/C)**	**Total sample (T/C)**	**Treatment group**	**Control group**	**Complications**	**Outcome**
Che XQ 2017	China	60.1 ± 5.1/59.2 ± 5.8	90 (45/45)	FA+VB12 + Metformin	Metformin	No	①
Guo CH 2018	China	58.65 ± 4.35/58.74 ± 5.01	66 (33/33)	FA + VB12 + Metformin	Metformin	No	①
Ju HB 2012	China	55.1 ± 9.7/54.8 ± 10.2	90 (43/47)	FA + VB12 + Metformin	Metformin	No	①
Li M 2018	China	62.11 ± 2.83/61.83 ± 2.76	90 (45/45)	FA + VB12 + Metformin	Metformin	No	①
Ma PT 2025	China	52.40 ± 2.01/52.32 ± 2.00	104 (52/52)	FA + VB12 + Metformin	Metformin	No	①
Ma XC 2017	China	52.7 ± 2.4/51.3 ± 2.4	100 (50/50)	FA + VB12 + Metformin	Metformin	Yes	①②④⑥⑦⑩
Shen CH 2017	China	63.9 ± 2.6/63.2 ± 2.5	102 (51/51)	FA + VB12 + Metformin	Metformin	Yes	①⑨⑩
Wang J 2018	China	56.3 ± 2.1/56.4 ± 2.0	80 (40/40)	FA + VB12 + Metformin	Metformin	Yes	①②④⑥⑦⑩
Wu YD 2015	China	60.45 ± 5.32	92 (46/46)	FA + VB12 + Metformin	Metformin	Yes	①
Xie CX 2015	China	56.4 ± 7.9/56.3 ± 8.2	200 (100/100)	FA + VB12 + Metformin	Metformin	No	①
Yang Y 2017	China	55.6 ± 6.1	65 (33/32)	FA + VB12 + Metformin	Metformin	No	①
Zhao XY 2020	China	56.18 ± 1.34/56.19 ± 1.39	108 (54/54)	FA + VB12 + Metformin	Metformin	No	①
Zhu XF 2017	China	66 ± 1.5/66 ± 1.8	114 (57/57)	FA + VB12 + Metformin	Metformin	No	①
Liang DT 2017	China	60.57 ± 2.34/60.32 ± 2.16	108 (54/54)	FA + VB12 + Metformin	Metformin	Yes	①②④⑩
Liu HB 2017	China	64.23 ± 1.33	62 (31/31)	FA + VB12 + Metformin	Metformin	Yes	①②④⑥⑩
Wang RZ 2017	China	61.7 + 4.2/61.1 ± 4.7	90 (45/45)	FA + VB12 + Metformin	Metformin	Yes	①②④⑥⑦⑩
Zhao WP 2018	China	53.08 ± 1.53/56.08 ± 1.73	200 (100/100)	FA + VB12 + Metformin	Metformin	No	①
Zhu F 2021	China	NR	80 (40/40)	FA + VB12 + Metformin	Metformin	No	①
Shen LJ 2017	China	48.9 ± 3.6	64 (32/32)	FA + VB12 + Metformin	Metformin	No	①
Su MZ 2017	China	58.9 ± 7.4/55.4 ± 6.5	40 (20/20)	FA + VB12 + Metformin	Metformin	Yes	①②③④⑤⑥⑩
Huang YR 2019	China	44.90 ± 6.30/46.10 ± 5.90	100 (50/50)	FA + VB12 + Metformin	Metformin	Yes	①③④⑥⑦⑧⑩
Li YJ 2012	China	NR	60 (30/30)	FA + VB12 + Metformin	Metformin	Yes	①②④⑥⑦⑩
Wei CS 2010	China	NR	40 (20/20)	FA + VB12 + CT	CT	No	①
Chen H 2012	China	53.1 ± 9.7	60 (30/30)	FA + VB12 + CT	CT	No	①
Guo PY 2011	China	56.88 ± 12.86	80 (40/40)	FA + VB12 + CT	CT	No	①
Satapathy S 2020	India	56.7 ± 9.6/54.2 ± 6.4	28 (19/19)	FA + VB12 + CT	CT	No	①
Levy Y 2009	Israel	60 ± 11/60 ± 9	30 (18/12)	FA + VB12 + B6 + CT	Placebo + CT	No	①
Mashavi M 2008	Israel	61.7 ± 6.5/60.1 ± 6.0	60 (28/29)	FA + VB12 + VB6 + Metformin	Placebo + Metformin	No	①
Fan SB 2010	China	58.72 ± 18.21	113 (61/52)	FA + VB6 + Metformin	Metformin	No	①

FA, folic acid; VB12, vitamin B12; VB6, vitamin B6; T/C, treatment group/control group; CT, conventional treatment; NR, not reported.

Complications: Homocysteine, Diabetic Kidney Disease, Diabetic Microangiopathy, Diabetic Peripheral Neuropathy, Myocardial Infarction, Cerebral Infarction, Coronary Heart Disease, Diabetic Foot, Hyperhomocysteinemia, Overall complications.

The primary efficacy outcome was serum HCY levels, whereas safety outcomes included the overall incidence and specific types of complications. All included RCTs involved complications such as diabetic nephropathy, microangiopathy, peripheral neuropathy, myocardial infarction, cerebral infarction, coronary artery disease, and diabetic foot.

### 2.4 Study quality assessment

All the studies included in this study were RCTs. Consequently, the Cochrane Collaboration's Risk of Bias 2.0 (RoB 2) tool was employed to evaluate the quality of the included studies (17). The assessment covered the following domains: the randomization process, deviations from the intended interventions, missing outcome data, measurement of the outcome, selection of the reported result, and overall bias. Two reviewers independently performed the quality assessment, and any disagreements were resolved through discussion or consultation with a third reviewer.

### 2.5 Statistical analysis

We conducted separate analyses for outcomes of different interventions. If the number of included studies was fewer than three, we performed a qualitative analysis only. To account for potential baseline imbalance, we extracted the mean and standard deviation of the change from baseline (post-intervention minus baseline) for subsequent analyses. For dichotomous variables, effect sizes were expressed as RRs. For continuous variables, effect sizes were reported as SMDs, with corresponding 95% CIs calculated. Given the anticipated heterogeneity, we used random-effects meta-analyses with the inverse variance method to pool RRs or SMDs. Knapp–Hartung adjustments were used to calculate CIs around the pooled effects. Between-study heterogeneity was estimated with *I*^2^, Cochran Q, and τ^2^ using the DerSimonian–Laird estimator. We conducted subgroup analyses based on the different comparison types to account for the potential influence of heterogeneity on the results. In addition, we separately assessed the summary RRs for total complications and specific complications. Publication bias was tested via funnel plot and Egger's test (18). If significant publication bias was detected, we further applied the trim-and-fill method to adjust the pooled results and assess the impact of publication bias. Given the low number of events for both overall and specific complications, we additionally conducted a sensitivity analysis to assess the robustness of the findings. Statistical analysis was conducted using R version 4.5.0 (R Foundation for Statistical Computing, Vienna, Austria) with the meta (version 8.1-0) package.

## 3 Results

### 3.1 Characteristics of the included studies

**Figure 1:**
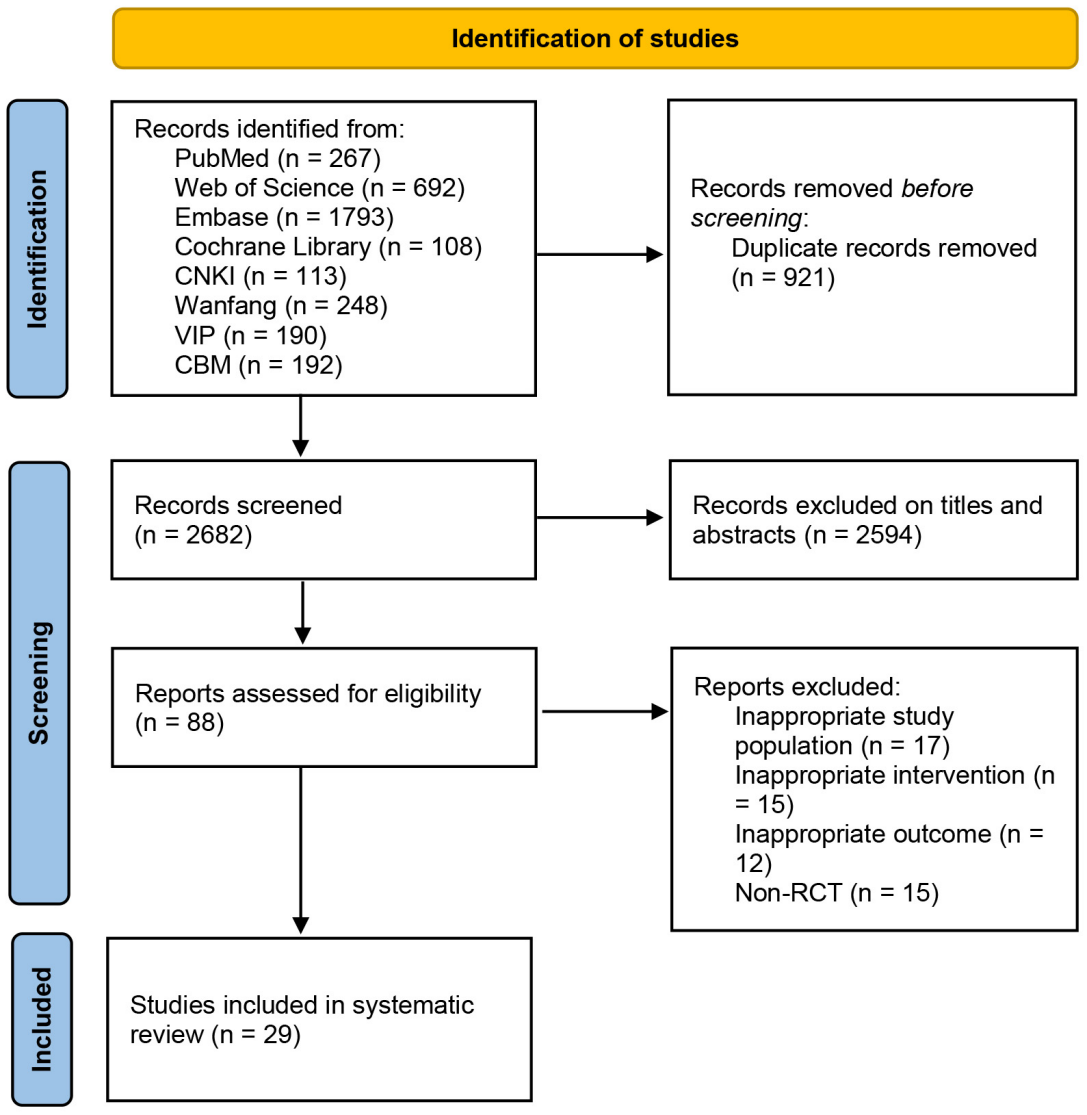
The flowchart illustrates the comprehensive process from the literature retrieval to study screening, with a specific focus on the selection procedure for RCTs. Source: Page et al. (16).

The systematic search conducted up to August 30, 2025, yielded 3,603 records. After removing 921 duplicates, we screened the titles and abstracts of 2,682 studies, excluding 2,594 that did not meet the eligibility criteria. The remaining 88 articles underwent a full-text assessment, from which 29 were ultimately included in the systematic review and meta-analysis. The detailed screening process is outlined in Figure 1.

The 29 included studies (Table 1) were categorized by intervention: 26 studies investigated folic acid plus vitamin B12 (19–44), two investigated folic acid plus vitamins B12 and B6 (45, 46), and one investigated folic acid plus vitamin B6 (47). All studies reported HCY levels. Among these, 9 studies (24–26, 32–34, 38–40) reported on the overall incidence of T2DM-related complications, with all nine also detailing the specific types of complications. Geographically, 26 studies were conducted in China, two in Israel, and one in India. The risk of bias assessment for the included RCTs, performed using the RoB 2 tool, is summarized in Supplementary Table S4.

### 3.2 Meta-analysis results

#### 3.2.1 HCY levels

**Figure 2:**
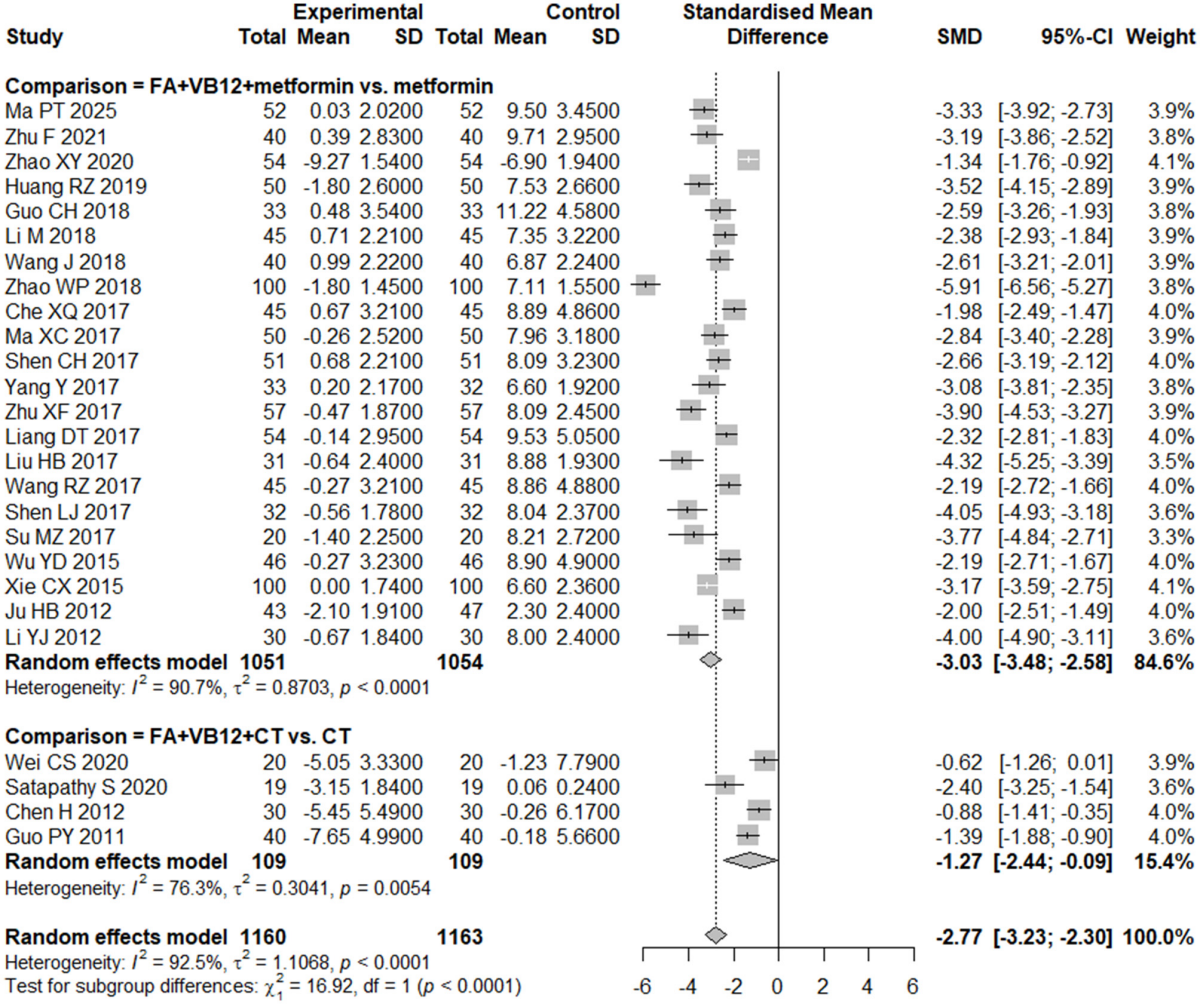
Forest plot of the effect of folic acid plus vitamin B12 on homocysteine levels.

The two studies reported that the HCY levels in the group receiving folic acid combined with vitamin B12 and B6 significantly decreased after the intervention compared to baseline, while the HCY levels in the placebo group showed a slight increase after the intervention compared to baseline. These two comparative results suggest that supplementation with folic acid, vitamin B12, and vitamin B6 may reduce HCY levels in patients with T2DM. One study added folic acid combined with vitamin B6 to the background therapy in the experimental group. Similarly, this study also reported a decrease in the mean change of HCY levels in the intervention group, while the metformin group showed an increase in the mean change of HCY levels. Given the number of eligible studies (*N* > 3), quantitative analyses were restricted to RCTs in which the intervention consisted solely of combined folic acid and vitamin B12 supplementation. Folic acid combined with vitamin B12 (SMD = −2.77, 95% CI [−3.23, −2.30], *P* < 0.0001) was effective in reducing HCY levels compared with those in the control group (Figure 2). Of concern was an observed level of heterogeneity (*I*^2^ = 92.5%) across the included studies. Publication bias was assessed by visualization of the funnel plot, and we observed evidence of bias on HCY (Supplementary Figure S1), and this was consistent with the findings of the Eggers regression test (*P* = 0.01). Similar estimators (SMD = −2.25, 95% CI [−2.78, −1.71]) were observed after adjustment with the trim-and-fill method (Supplementary Figure S2). In addition, we conducted subgroup analyses restricted to studies with comparable interventions and comparators. The results showed that, compared with metformin alone, combined folic acid plus vitamin B12 supplementation on top of metformin therapy significantly lowered HCY levels (Figure 2). Likewise, folic acid plus vitamin B12 added to usual care also produced a significant reduction in HCY relative to usual care alone (Figure 2).

#### 3.2.2 Incidence of complications

**Figure 3:**
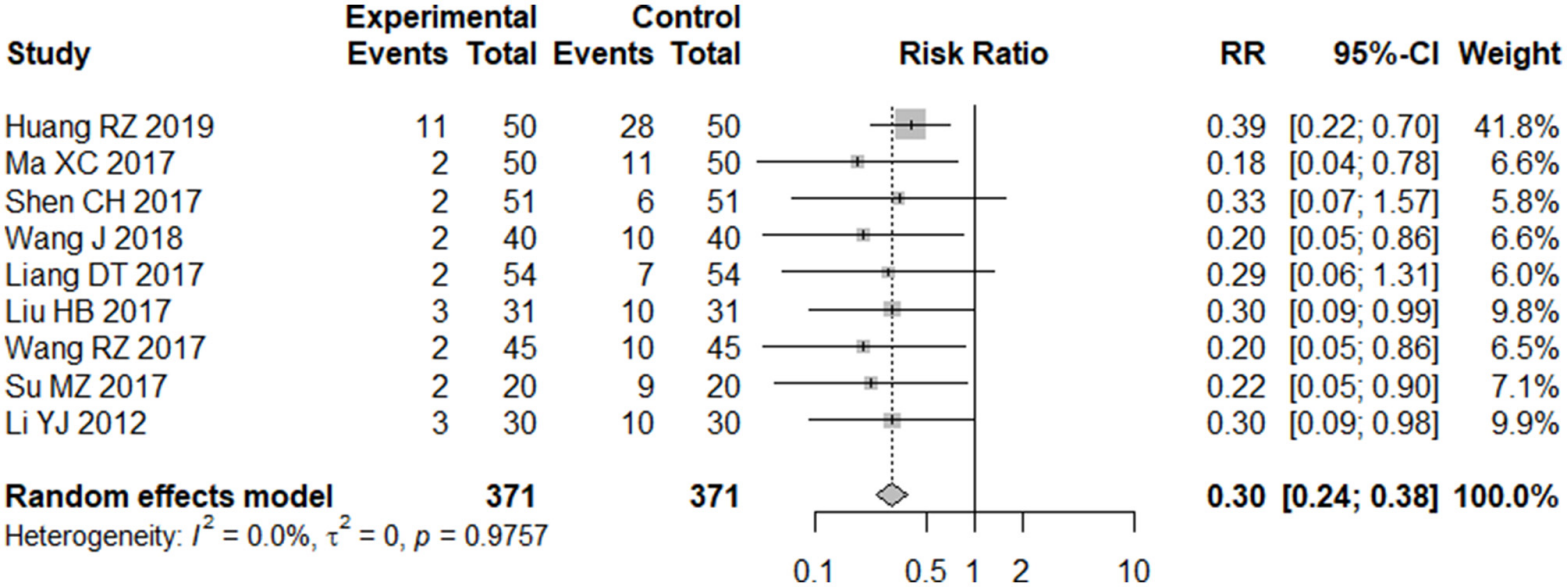
Forest plot of the effect of folic acid plus vitamin B12 on overall complications.

Nine RCTs evaluating overall complications compared folic acid and vitamin B12 plus metformin vs. metformin alone. Folic acid combined with vitamin B12 significantly reduced the incidence of complications in T2DM patients compared with controls (RR = 0.30, 95% CI [0.24, 0.38], *P* < 0.0001) (Figure 3). The heterogeneity test confirmed homogeneity among studies (*I*^2^ = 0%, *P* = 0.98). Publication bias was assessed by visualization of the funnel plot, and we observed evidence of bias on incidence of complications (Supplementary Figure S3), and this was consistent with the findings of the Eggers regression test (*P* < 0.01). Similar results (RR = 0.36, 95% CI [0.28, 0.45]) were observed after adjustment with the trim-and-fill method (Supplementary Figure S4). The sensitivity analysis results indicated the robustness of the current findings (Supplementary Figure S5).

**Figure 4:**
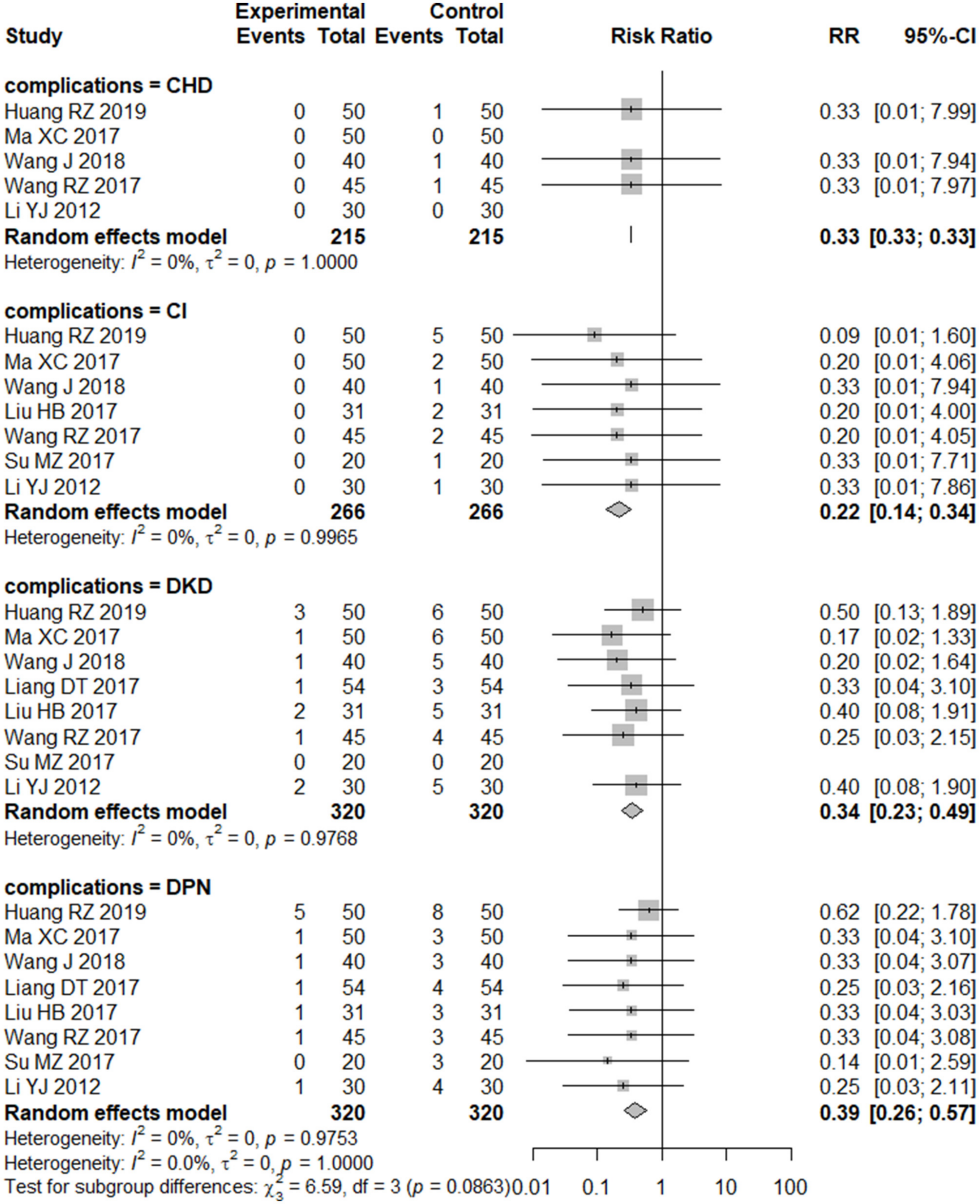
Forest plot of the effect of folic acid plus vitamin B12 on specific complications.

Further analyses showed that folic acid plus vitamin B12 supplementation significantly reduced the risk of specific diabetic complications, including diabetic kidney disease, diabetic peripheral neuropathy, cerebral infarction, and coronary heart disease (Figure 4). The sensitivity analysis results indicated the robustness of the current findings (Supplementary Figures S6–S9).

## 4 Discussion

Our analysis suggests that supplementation with folic acid and vitamin B12 is associated not only with a significant reduction in serum HCY levels but also with a potential reduction in the risk of overall diabetes-related complications. However, this conclusion should be interpreted with caution due to the high heterogeneity observed across the included studies. Although supplementation showed potential benefits for complications such as diabetic nephropathy, peripheral neuropathy, and cerebral infarction, the evidence remains inconclusive due to limited data.

HCY is a sulfur-containing amino acid and an intermediate metabolite produced during methionine demethylation (48). Folic acid (vitamin B9) and its active form, 5-methyltetrahydrofolate (5-MTHF), are key regulators of HCY metabolism. Folic acid facilitates the remethylation of HCY into methionine via the methionine-folate cycle, reducing elevated HCY levels (49). One-carbon metabolism, involving folic acid, vitamin B6, and vitamin B12, supports DNA synthesis and methylation through one-carbon unit transfer (50). These vitamins act synergistically to maintain normal HCY metabolism, whereas deficiencies disrupt one-carbon metabolism, leading to HCY accumulation (51). Folic acid exhibits multifaceted beneficial effects in metabolic regulation. In medicine, strategies exploiting the folic acid receptor and engineering key sites like cysteine have been validated in oncology. In public health, folic acid intake mitigates PFAS exposure-induced elevations in triglyceride levels, thereby helping to reduce the risks of hyperlipidemia and atherosclerosis (52, 53). Disrupted gut microbiota and metabolic disorders are key contributors to diabetes development. They also play an irreplaceable role in immune regulation and inflammatory responses. A systematic review indicates that polyphenols can help regulate gut microbiota homeostasis. Specifically, flavonoids enhance intestinal barrier function by modulating intracellular molecular mechanisms (54). A review of the Chinese herb Astragali Radix indicates that its abundant polysaccharides can reduce oxidative and intestinal inflammatory stress. This action helps restore the gut barrier and delay IR, thereby achieving glycemic control (55). The attenuation of cellular inflammation and myocardial fibrosis by Syringaresinol, via modulation of the TGF-β/Smad pathway, delays the progression of cardiovascular complications in T2DM (56). Beyond direct vitamin supplementation, dietary interventions that modulate the gut microbiota have also been shown to help improve T2DM and its complications. A study by Li et al. (57) demonstrated that polyphenol-rich Mulberry Leaf Fu tea remodels the gut microbiome in diabetic mice. It significantly promoted the growth of beneficial bacteria, such as Lactobacillus and Bifidobacterium. Consequently, the treatment improved insulin levels and reduced tissue cell damage, providing experimental evidence for managing T2DM via microbial intervention. Another investigation demonstrated that sequentially fermented, dealcoholized apple juice modulates the gut microbiota composition. This modulation improved lipid profiles and optimized B vitamins metabolism, ultimately reducing HCY levels in patients with T2DM. These findings offer a novel dietary intervention strategy for preventing associated metabolic disorders and vascular complications (58).

Genetic factors play a critical role in the pathogenesis of DM. The allelic and recessive genotypic models of the ICAM-1 rs5498 polymorphism have been associated with susceptibility to DM in Asian populations (59). Previous meta-analyses have explored the effects of folic acid and B vitamins on various populations and clinical indicators. For example, Y Miao et al. reported that folic acid and B vitamins reduce CVD risk but do not significantly affect HCY levels (60). Zhang et al. (61) demonstrated that supplementation significantly lowers HCY concentrations in metformin-treated patients. Mokgalaboni et al. (62) reported that folic acid supplementation reduces HCY levels and CVD risk in T2DM patients. Our findings align with these studies but specifically focus on T2DM patients. Forest plot analyses revealed significant reductions in HCY levels among T2DM patients receiving folic acid combined with vitamin B12, which was validated by subgroup analyses. The combination of folic acid with vitamin B12 and B6 has a stronger effect on reducing HCY levels in T2DM patients with peripheral neuropathy.

The evidence indicates that B vitamins play a critical role in HCY metabolism (63). Deficiencies in these vitamins increase total HCY levels, contributing to CVD development (64). This systematic meta-analysis examines the impact of folic acid and B vitamins on the incidence of complications in T2DM patients, extending beyond CVD-focused research. Our findings demonstrate that supplementation significantly reduces the incidence of complications, particularly diabetic nephropathy and peripheral neuropathy. Observational studies have linked elevated plasma tHcy levels to diabetic nephropathy, peripheral neuropathy, and vascular diseases (65). A US-based multicenter RCT revealed a greater decline in the glomerular filtration rate in the B vitamins group than in the placebo group (66). Additionally, in children with T1DM, B vitamins improve glycemic control and renal function by lowering HCY levels (67).

Studies have established a significant association between diabetic retinopathy and diabetic peripheral neuropathy (68). HCY induces neuronal cell death by stimulating N-methyl-D-aspartate (NMDA) receptors. Moore et al.'s (69) animal experiments demonstrated that elevated HCY and glutamate enhance excitotoxic damage to retinal ganglion cells. Elevated plasma tHcy levels are correlated with peripheral neuropathy onset and serve as an independent risk factor (70). Peng et al. (71) demonstrated that KAT2A activates SAT2 transcription via H3K79succ, thereby promoting ferroptosis and ultimately aggravating kidney damage. Long-term metformin use impairs vitamin B12 absorption and elevates HCY and methylmalonic acid (MMA) levels, inducing neurotoxicity via oxidative stress and mitochondrial dysfunction (72). The retrospective study by Ruotong Yang et al. highlighted increased hospitalization risk for peripheral neuropathy in T2DM patients receiving metformin treatment, which was positively correlated with daily dosage (73). Supplementation with folic acid and B vitamins improves HCY metabolism, reduces oxidative stress and inflammation, and delays nephropathy and neuropathy progression in diabetes.

This study has several notable limitations that warrant consideration. First, the meta-analysis results indicated that folic acid combined with vitamin B12 supplementation can significantly reduce HCY levels in patients with T2DM, significant heterogeneity was also observed. Given that the vast majority of studies did not report detailed information on the treatment regimen (dosage, frequency, duration, and HCY assay methods), we were unable to conduct further subgroup analyses or meta-regression to explore the sources of heterogeneity. In addition, since different studies measured HCY in different units (e.g., mmol/L, nmol/L), we used effect sizes standardized to eliminate unit differences. Nevertheless, we still observed large estimated values (SMD = −2.77), reflecting high heterogeneity among the original studies. Therefore, our current findings should be interpreted with caution, and high-quality, large-scale RCTs are urgently needed in the future. Second, the majority of the included RCTs have an uncertain risk of bias, which may affect the reliability of our conclusions. Methodological concerns were identified, including unclear descriptions of randomization procedures and bias in measurement of the outcome. These factors collectively introduce potential biases and weaken the robustness of the evidence. Notably, the incidence of complications in patients with T2DM reported in primary study was low, which may reflect inadequate diagnosis and management. Moreover, the vast majority of primary studies did not explicitly define the diagnostic criteria for related complications in the methodology section, but merely reported the number of complication events in the results. This may introduce measurement bias. Future research should clearly and explicitly define the diagnostic criteria for various types of complications, and consistently apply the same diagnostic criteria to both the experimental and control groups. Third, significant publication bias was identified, necessitating cautious interpretation. The pooled effect size adjusted by the trim-and-fill method remained consistent with the primary results; however, due to the high heterogeneity, this adjusted estimate must be regarded as exploratory. Finally, due to the limited number of RCTs, our study was unable to further quantitatively evaluate the intervention effects of folic acid combined with vitamin B12 and B6 supplementation, as well as folic acid combined with vitamin B6 alone. Future large-scale, high-quality RCTs are still needed to further explore the effects of folic acid supplementation in combination with vitamin B12 and B6.

## 5 Conclusion

This study demonstrates that, in patients with T2DM, supplementation with folic acid plus vitamin B12 on top of routine glucose-lowering therapy significantly lowers serum HCY levels and reduces the overall incidence of complications. However, the high heterogeneity across studies and the low number of outcome events reported in the primary studies necessitate cautious interpretation of these preliminary findings. Additional large-scale, high-quality RCTs are required to validate our results. Limited evidence also suggests a potential benefit of folic acid combined with vitamin B6 in reducing serum HCY in these patients, and such studies are urgently needed.

## Data Availability

The original contributions presented in the study are included in the article/Supplementary material, further inquiries can be directed to the corresponding authors.
