# Supplementary material for: Effects of folic acid with vitamin B12/vitamin B6 intervention on serum homocysteine metabolism and complications in patients with type 2 diabetes: a systematic review and meta-analysis of randomized controlled trials
Source: Front Nutr. 2025 Nov 28;12:1701310. doi: 10.3389/fnut.2025.1701310 (PMC12698435; doi:10.3389/fnut.2025.1701310)
Supplement: Supplementary file 1 [file Table_1.docx]

Effects of Folic Acid with Vitamin B12/Vitamin B6 Intervention on Serum Homocysteine Metabolism and Complications in Patients with Type 2 Diabetes: A Systematic Review and Meta-Analysis of Randomized Controlled Trials

**Table of contents**

| **Table S1.** Preferred Reporting Items for Systematic reviews and Meta-Analysis (PRISMA) 2020 Checklist. |
| --- |
| **Table S2.** Deviations from the PROSPERO Registered Protocol. |
| **Table S3.** Search strategy (From database inception to August 30, 2025). |
| **Table S4.** Risk of Bias Assessment for Included Randomized Controlled Trials Using the Cochrane RoB 2 Tool. |
| **Figure S1.** Funnel plot of homocysteine levels |
| **Figure S2.** Funnel plot of homocysteine levels after trim-and-fill adjustment |
| **Figure S3.** Funnel plot of overall complication rates |
| **Figure S4.** Funnel plot of overall complication rates after trim-and-fill adjustment |
| **Figure S5.** Sensitivity analysis of overall complication rates |
| **Figures S6-S9.** Sensitivity analysis of specific complication rates |

| **Table S1. Preferred Reporting Items for Systematic reviews and Meta-Analysis (PRISMA) 2020 Checklist.** | | | |
| --- | --- | --- | --- |
| **Section and Topic** | **Item #** | **Checklist item** | **Location where item is reported** |
| **TITLE** | | |  |
| Title | 1 | Identify the report as a systematic review. | Title |
| **ABSTRACT** | | |  |
| Abstract | 2 | See the PRISMA 2020 for Abstracts checklist. | Abstract |
| **INTRODUCTION** | | |  |
| Rationale | 3 | Describe the rationale for the review in the context of existing knowledge. | Introduction |
| Objectives | 4 | Provide an explicit statement of the objective(s) or question(s) the review addresses. | Introduction |
| **METHODS** | | |  |
| Eligibility criteria | 5 | Specify the inclusion and exclusion criteria for the review and how studies were grouped for the syntheses. | Eligibility criteria |
| Information sources | 6 | Specify all databases, registers, websites, organisations, reference lists and other sources searched or consulted to identify studies. Specify the date when each source was last searched or consulted. | Search strategy |
| Search strategy | 7 | Present the full search strategies for all databases, registers and websites, including any filters and limits used. | Search strategy  Table S3 |
| Selection process | 8 | Specify the methods used to decide whether a study met the inclusion criteria of the review, including how many reviewers screened each record and each report retrieved, whether they worked independently, and if applicable, details of automation tools used in the process. | Study selection and data extraction |
| Data collection process | 9 | Specify the methods used to collect data from reports, including how many reviewers collected data from each report, whether they worked independently, any processes for obtaining or confirming data from study investigators, and if applicable, details of automation tools used in the process. | Study selection and data extraction |
| Data items | 10a | List and define all outcomes for which data were sought. Specify whether all results that were compatible with each outcome domain in each study were sought (e.g. for all measures, time points, analyses), and if not, the methods used to decide which results to collect. | Study selection and data extraction |
|  | 10b | List and define all other variables for which data were sought (e.g. participant and intervention characteristics, funding sources). Describe any assumptions made about any missing or unclear information. | Study selection and data extraction |
| Study risk of bias assessment | 11 | Specify the methods used to assess risk of bias in the included studies, including details of the tool(s) used, how many reviewers assessed each study and whether they worked independently, and if applicable, details of automation tools used in the process. | Quality assessment |
| Effect measures | 12 | Specify for each outcome the effect measure(s) (e.g. risk ratio, mean difference) used in the synthesis or presentation of results. | Data analysis |
| Synthesis methods | 13a | Describe the processes used to decide which studies were eligible for each synthesis (e.g. tabulating the study intervention characteristics and comparing against the planned groups for each synthesis (item #5)). | Data analysis |
|  | 13b | Describe any methods required to prepare the data for presentation or synthesis, such as handling of missing summary statistics, or data conversions. | Data analysis |
|  | 13c | Describe any methods used to tabulate or visually display results of individual studies and syntheses. | Data analysis |
|  | 13d | Describe any methods used to synthesize results and provide a rationale for the choice(s). If meta-analysis was performed, describe the model(s), method(s) to identify the presence and extent of statistical heterogeneity, and software package(s) used. | Data analysis |
|  | 13e | Describe any methods used to explore possible causes of heterogeneity among study results (e.g. subgroup analysis, meta-regression). | Data analysis |
|  | 13f | Describe any sensitivity analyses conducted to assess robustness of the synthesized results. | Data analysis |
| Reporting bias assessment | 14 | Describe any methods used to assess risk of bias due to missing results in a synthesis (arising from reporting biases). | Data analysis |
| Certainty assessment | 15 | Describe any methods used to assess certainty (or confidence) in the body of evidence for an outcome. | NA |
| **RESULTS** | | |  |
| Study selection | 16a | Describe the results of the search and selection process, from the number of records identified in the search to the number of studies included in the review, ideally using a flow diagram. | Description of studies  Fig. 1 |
|  | 16b | Cite studies that might appear to meet the inclusion criteria, but which were excluded, and explain why they were excluded. | Description of studies |
| Study characteristics | 17 | Cite each included study and present its characteristics. | Table 1 |
| Risk of bias in studies | 18 | Present assessments of risk of bias for each included study. | Table S4 |
| Results of individual studies | 19 | For all outcomes, present, for each study: (a) summary statistics for each group (where appropriate) and (b) an effect estimate and its precision (e.g. confidence/credible interval), ideally using structured tables or plots. | Meta-Analysis and Sensitivity Analysis |
| Results of syntheses | 20a | For each synthesis, briefly summarise the characteristics and risk of bias among contributing studies. | Meta-Analysis and Sensitivity Analysis |
|  | 20b | Present results of all statistical syntheses conducted. If meta-analysis was done, present for each the summary estimate and its precision (e.g. confidence/credible interval) and measures of statistical heterogeneity. If comparing groups, describe the direction of the effect. | Meta-Analysis and Sensitivity Analysis |
|  | 20c | Present results of all investigations of possible causes of heterogeneity among study results. | Subgroup analysis |
|  | 20d | Present results of all sensitivity analyses conducted to assess the robustness of the synthesized results. | Fig. S5-S9 |
| Reporting biases | 21 | Present assessments of risk of bias due to missing results (arising from reporting biases) for each synthesis assessed. | Publication bias  Fig. S1-S4 |
| Certainty of evidence | 22 | Present assessments of certainty (or confidence) in the body of evidence for each outcome assessed. | NA |
| **DISCUSSION** | | |  |
| Discussion | 23a | Provide a general interpretation of the results in the context of other evidence. | Discussion |
|  | 23b | Discuss any limitations of the evidence included in the review. | Limitations |
|  | 23c | Discuss any limitations of the review processes used. | Limitations |
|  | 23d | Discuss implications of the results for practice, policy, and future research. | Conclusion |
| **OTHER INFORMATION** | | |  |
| Registration and protocol | 24a | Provide registration information for the review, including register name and registration number, or state that the review was not registered. | Methods |
|  | 24b | Indicate where the review protocol can be accessed, or state that a protocol was not prepared. | Methods |
|  | 24c | Describe and explain any amendments to information provided at registration or in the protocol. | Methods |
| Support | 25 | Describe sources of financial or non-financial support for the review, and the role of the funders or sponsors in the review. | Statements and Declarations |
| Competing interests | 26 | Declare any competing interests of review authors. | Statements and Declarations |
| Availability of data, code and other materials | 27 | Report which of the following are publicly available and where they can be found: template data collection forms; data extracted from included studies; data used for all analyses; analytic code; any other materials used in the review. | Statements and Declarations |

Table S2. Deviations from the PROSPERO Registered Protocol.

| As pre-specified in the registered protocol | As described in this study | Reasons |
| --- | --- | --- |
| PRISMA 2009 (Liberati 2009) | PRISMA 2020 statement | Taking into account the reviewers' opinions, use the updated standards to conduct the quality assessment. Improved the methodological rigor and reporting quality of the research |
| Cochrane RoB tool (2011) | Cochrane Risk of Bias 2 (RoB 2) tool | Taking into account the reviewers' opinions, use the updated standards to conduct the quality assessment. Improved the methodological rigor and reporting quality of the research |
| DerSimonian-Laird (DL) estimator for τ² under a random-effects model | Knapp-Hartung adjustments were used to calculate CIs around the pooled effects. | Taking into account the opinions of the reviewers, it has been upgraded to a complete set of strategy combinations, enhancing its robustness. |

Table S3. Search strategy (From database inception to August 30, 2025).

| Databases | Query | Results |
| --- | --- | --- |
| PubMed | #1: "diabetes mellitus, type 2"[MeSH Terms] OR "type 2 diabetes mellitus"[All Fields] OR "T2DM"[All Fields] OR ("diabetes mellitus, type 2"[MeSH Terms] OR "type 2 diabetes mellitus"[All Fields] OR "type 2 diabetes"[All Fields]) OR ("diabetes mellitus, type 2"[MeSH Terms] OR "type 2 diabetes mellitus"[All Fields] OR ("type"[All Fields] AND "ii"[All Fields] AND "diabetes"[All Fields] AND "mellitus"[All Fields]) OR "type ii diabetes mellitus"[All Fields]) OR ("diabetes mellitus, type 2"[MeSH Terms] OR "type 2 diabetes mellitus"[All Fields] OR "diabetes mellitus type 2"[All Fields]) | 276,228 |
|  | #2: "folic acid"[Supplementary Concept] OR "folic acid"[All Fields] OR "folic acid"[MeSH Terms] OR ("folic"[All Fields] AND "acid"[All Fields]) OR ("folic acid"[Supplementary Concept] OR "folic acid"[All Fields] OR "folate"[All Fields] OR "folic acid"[MeSH Terms] OR ("folic"[All Fields] AND "acid"[All Fields]) OR "folates"[All Fields]) OR ("folic acid"[Supplementary Concept] OR "folic acid"[All Fields] OR "folacin"[All Fields] OR "folic acid"[MeSH Terms] OR ("folic"[All Fields] AND "acid"[All Fields]) OR "folate"[All Fields] OR "folates"[All Fields]) OR ("folic acid"[Supplementary Concept] OR "folic acid"[All Fields] OR "vitamin b9"[All Fields] OR "folic acid"[MeSH Terms] OR ("folic"[All Fields] AND "acid"[All Fields]) OR ("vitamin"[All Fields] AND "b9"[All Fields])) OR ("folic acid"[Supplementary Concept] OR "folic acid"[All Fields] OR "vitamin m"[All Fields] OR "folic acid"[MeSH Terms] OR ("folic"[All Fields] AND "acid"[All Fields])) OR ("5 methyltetrahydrofolate"[Supplementary Concept] OR "5 methyltetrahydrofolate"[All Fields] OR "5 methyltetrahydrofolate"[All Fields]) OR ("folic acid"[Supplementary Concept] OR "folic acid"[All Fields] OR "pteroylglutamic acid"[All Fields] OR "folic acid"[MeSH Terms] OR ("folic"[All Fields] AND "acid"[All Fields]) OR ("pteroylglutamic"[All Fields] AND "acid"[All Fields])) | 75,454 |
|  | #3: "vitamin b 12"[Supplementary Concept] OR "vitamin b 12"[All Fields] OR "vitamin b12"[All Fields] OR "vitamin b 12"[MeSH Terms] OR ("vitamin"[All Fields] AND "b12"[All Fields]) OR ("cobalamine"[All Fields] OR "cobalamines"[All Fields] OR "vitamin b 12"[Supplementary Concept] OR "vitamin b 12"[All Fields] OR "cobalamin"[All Fields] OR "vitamin b 12"[MeSH Terms] OR "cobalamins"[All Fields]) OR ("mecobalamin"[Supplementary Concept] OR "mecobalamin"[All Fields] OR "methylcobalamin"[All Fields] OR "methylcobalamine"[All Fields]) OR ("vitamin b 6"[Supplementary Concept] OR "vitamin b 6"[All Fields] OR "vitamin b6"[All Fields] OR "vitamin b 6"[MeSH Terms] OR ("vitamin"[All Fields] AND "b6"[All Fields])) OR ("pyridoxal phosphate"[Supplementary Concept] OR "pyridoxal phosphate"[All Fields] OR "pyridoxal 5 phosphate"[All Fields] OR "pyridoxal phosphate"[MeSH Terms] OR ("pyridoxal"[All Fields] AND "phosphate"[All Fields])) OR ("pyridoxine"[Supplementary Concept] OR "pyridoxine"[All Fields] OR "pyridoxin"[All Fields] OR "pyridoxine"[MeSH Terms] OR "pyridoxines"[All Fields]) | 64,225 |
|  | #4: #1 AND #2 AND #3 | 267 |
| Web of Science | #1: TS=(type 2 diabetes mellitus OR T2DM OR type 2 diabetes OR type II diabetes mellitus OR diabetes mellitus type 2) | 577,768 |
|  | #2: TS=(folic acid OR folate OR folacin OR vitamin B9 OR vitamin M OR 5-methyltetrahydrofolate OR pteroylglutamic acid) | 199,421 |
|  | #3: TS=(vitamin B6 OR pyridoxal 5′-phosphate OR pyridoxine OR vitamin B12 OR cobalamin OR methylcobalamin) | 111,951 |
|  | #4: #1 AND #2 AND #3 | 692 |
| Embase | #1: type AND 2 AND diabetes AND mellitus OR t2dm OR (type AND 2 AND diabetes) OR (type AND ii AND diabetes AND mellitus) OR (diabetes AND mellitus AND type AND 2) | 568,807 |
|  | #2: folic AND acid OR folate OR folacin OR (vitamin AND b9) OR (vitamin AND m) OR '5 methyltetrahydrofolate' OR (pteroylglutamic AND acid) | 315,915 |
|  | #3: vitamin AND b6 OR (pyridoxal AND '5′ phosphate') OR pyridoxine OR (vitamin AND b12) OR cobalamin OR methylcobalamin | 81,098 |
|  | #4: #1 AND #2 AND #3 | 1,793 |
| Cochrane Library | #1: (type 2 diabetes mellitus OR T2DM OR type 2 diabetes OR type II diabetes mellitus OR diabetes mellitus type 2):ti,ab,kw | 68,701 |
|  | #2: (folic acid OR folate OR folacin OR vitamin B9 OR vitamin M OR methyltetrahydrofolate OR pteroylglutamic acid):ti,ab,kw | 11,346 |
|  | #3: (vitamin B6 OR pyridoxal OR pyridoxine OR vitamin B12 OR cobalamin OR methylcobalamin):ti,ab,kw | 4,444 |
|  | #4: #1 AND #2 AND #3 | 108 |
| CNKI | （主题: 2型糖尿病 + 消渴 + T2DM) AND (主题: 叶酸 + 维生素B9 + 维生素M) AND (主题: 维生素B6 + 吡哆醇 + 维生素B12 + 甲钴胺) | 113 |
| Wanfang | 主题:(2型糖尿病 OR 消渴 OR T2DM) and 主题:(叶酸 OR 维生素B9 OR 维生素M) and 主题:(维生素B6 OR 吡哆醇 OR 维生素B12 OR 甲钴胺)) | 248 |
| VIP | ((((任意字段=2型糖尿病 OR 任意字段=消渴) OR 任意字段=T2DM) AND ((任意字段=叶酸 OR 任意字段=维生素B9) OR 任意字段=维生素M)) AND (((任意字段=维生素B6 OR 任意字段=吡哆醇) OR 任意字段=维生素B12) OR 任意字段=甲钴胺)) | 190 |
| CBM | ("2型糖尿病"[全部字段:智能] OR "消渴"[全部字段:智能] OR "T2DM"[全部字段:智能]) AND ("叶酸"[全部字段:智能] OR "维生素B9"[全部字段:智能] OR "维生素M"[全部字段:智能]) AND ("维生素B6"[全部字段:智能] OR "吡哆醇"[全部字段:智能] OR "维生素B12"[全部字段:智能] OR "甲钴胺"[全部字段:智能]) | 192 |

| Table S4. Risk of Bias Assessment for Included Randomized Controlled Trials Using the Cochrane RoB 2 Tool. | | | | | | |
| --- | --- | --- | --- | --- | --- | --- |
| Study ID | D1 | D2 | D3 | D4 | D5 | Overall |
| Che XQ 2017 | 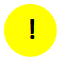 | 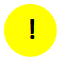 | 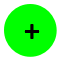 | 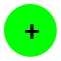 | 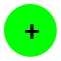 | 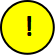 |
| Guo CH 2018 | 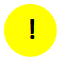 | 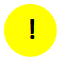 | 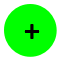 | 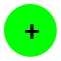 | 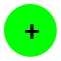 | 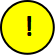 |
| Ju HB 2012 | 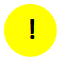 | 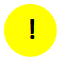 | 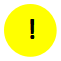 | 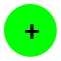 | 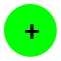 | 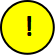 |
| Li M 2018 | 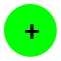 | 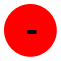 | 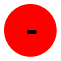 | 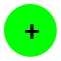 | 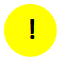 | 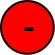 |
| Ma PT 2025 | 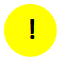 | 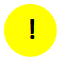 | 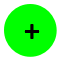 | 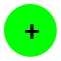 | 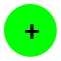 | 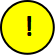 |
| Ma XC 2017 | 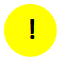 | 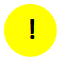 | 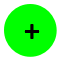 | 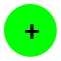 | 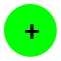 | 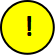 |
| Shen CH 2017 | 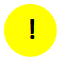 | 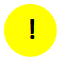 | 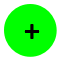 | 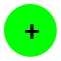 | 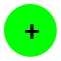 | 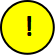 |
| Wang J 2018 | 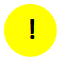 | 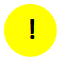 | 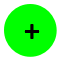 | 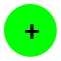 | 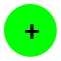 | 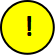 |
| Wu YD 2015 | 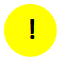 | 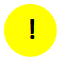 | 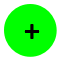 | 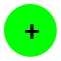 | 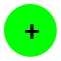 | 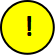 |
| Xie CX 2015 | 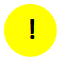 | 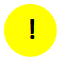 | 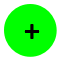 | 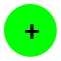 | 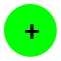 | 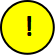 |
| Yang Y 2017 | 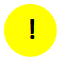 | 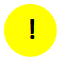 | 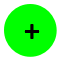 | 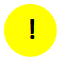 | 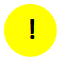 | 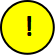 |
| Zhao XY 2020 | 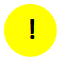 | 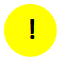 | 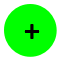 | 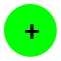 | 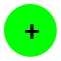 | 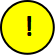 |
| Zhu XF 2017 | 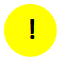 | 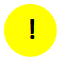 | 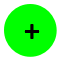 | 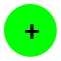 | 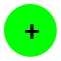 | 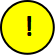 |
| Liang DT 2017 | 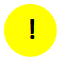 | 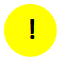 | 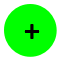 | 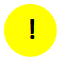 | 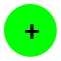 | 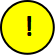 |
| Liu HB 2017 | 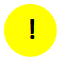 | 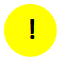 | 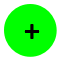 | 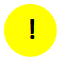 | 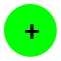 | 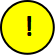 |

| Study ID | D1 | D2 | D3 | D4 | D5 | Overall |
| --- | --- | --- | --- | --- | --- | --- |
| Wang RZ 2017 | 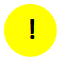 | 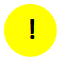 | 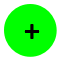 | 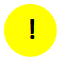 | 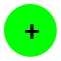 | 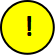 |
| Zhao WP 2018 | 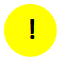 | 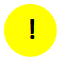 | 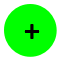 | 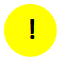 |  |  |
| Zhu F 2021 |  |  |  |  |  |  |
| Shen LJ 2017 |  |  |  |  |  |  |
| Su MZ 2017 |  |  |  |  |  |  |
| Huang YR 2019 |  |  |  |  |  |  |
| Li YJ 2012 |  |  |  |  |  |  |
| Wei CS 2010 |  |  |  |  |  |  |
| Chen H 2012 |  |  |  |  |  |  |
| Guo PY 2011 |  |  |  |  |  |  |
| Satapathy S 2020 |  |  |  |  |  |  |
| Levy Y 2009 |  |  |  |  |  |  |
| Mashavi M 2008 |  |  |  |  |  |  |
| Fan SB 2010 |  |  |  |  |  |  |

D1: Randomisation process; D2: Deviations from the intended interventions; D3: Missing outcome data; D4: Measurement of the outcome; D5: Selection of the reported result; Low risk; Some concerns; High risk.

Figure S1. Funnel plot of homocysteine levels

Figure S2. Funnel plot of homocysteine levels after trim-and-fill adjustment

Figure S3. Funnel plot of overall complication rates

Figure S4. Funnel plot of overall complication rates after trim-and-fill adjustment

Figure S5. Sensitivity analysis of overall complication rates

Figure S6. Sensitivity analysis of specific complication rates (coronary heart disease)

Figure S7. Sensitivity analysis of specific complication rates (cerebral infarction)

Figure S8. Sensitivity analysis of specific complication rates (diabetic kidney disease)

Figure S9. Sensitivity analysis of specific complication rates (diabetic peripheral neuropathy)
